# Global interest in vaccines during the COVID‐19 pandemic: Evidence from Google Trends

**DOI:** 10.1016/j.jvacx.2022.100152

**Published:** 2022-03-11

**Authors:** Aida Khakimova, Leila Abdollahi, Oleg Zolotarev, Fakher Rahim

**Affiliations:** a Department of Development of Scientific and Innovation Activities, Russian New University, Moscow, Russia; b Department of Medical Library and Information Scince, School of Health Managment and Information Sciences, Iran University of Medical Sciences, Tehran, Iran; c Department of Information Systems in Economics and Management, Russian New University, Moscow, Russia; d Metabolomics and Genomics Research Center, Tehran University of Medical Sciences, Tehran, Iran; e Health Research Institute, Thalassemia and Hemoglobinopathy Research Centre, Ahvaz Jundishapur University of Medical Sciences, Ahvaz, Iran

**Keywords:** Coronavirus vaccine, Corona vaccine, COVID-19 vaccine, Pfizer vaccine, AstraZeneca vaccine, Sputnik v, Vaccination

## Abstract

COVID-19 (coronavirus disease 2019) vaccines have become available; now, everyone has the opportunity to get vaccinated. We used Google Trends (GT) data to assess the global public interest in COVID-19 vaccines during the pandemic. For the analysis, a period of 17 months was chosen (from Jan 19, 2020, to Jul 04, 2021). Interest in user queries was tracked by keywords (corona vaccine, COVID-19 vaccine development, Sputnik v, Pfizer vaccine, AstraZeneca vaccine, etc.). The geographic analysis of queries was also carried out. The interest of users in the vaccine is significantly increasing. It is focused on the side effects of vaccines, and users pay attention to vaccines’ developers from different countries. The correlation between the scientific publications devoted to vaccine development and such requests of users on the internet is absent. This study shows that internet search patterns can be used to gauge public attitudes towards coronavirus vaccination. Safety concerns consistently high follow an interest in vaccine side effects. This data can be used to track and predict attitudes towards vaccination of populations from COVID-19 in different countries before global vaccination becomes available to help mitigate the adverse effects of the pandemic.

## Introduction

Severe acute respiratory syndrome coronavirus (SARS-CoV-2) is a new human virus that causes COVID-19 (coronavirus disease 2019). The human population does not have the necessary immunity to protect themselves against the transmission of the virus and disease [1]. To be approved, COVID-19 vaccines become available, and everyone can get vaccinated, including children in some countries. For pregnant women, COVID-19 vaccines are recommended with care [2]. Initially, when only a limited number of vaccines were available, vaccination was prioritized following procedures proposed by the World Health Organization (WHO) [3]. Vaccination for COVID-19 was primarily recommended for residents of care centers and nursing homes, healthcare providers, especially those in intensive care units (ICUs), and the elderly as well as the elderly in vulnerable groups [4]. Vaccines are now broadly accessible and do not need an appointment or a specific brand; even those aged 12 and over can currently receive their free COVID-19 vaccination [1], [5]. Thus, COVID-19 vaccine recommendations now differ widely based on accessibility and country-specific vaccination campaigns [6].

Google Trends (GT) is one of the essential tools for cataloguing internet queries, and this indexed information is available [7]. The large database provided by GT can reveal patterns in the search for information at the population level, allowing the development of targeted information for the public [8], [9].

The use of GT in various diseases outbreak is nothing new and has previously been used in infectious diseases such as influenza and Zika virus [10], [11], [12], [13]. Interestingly, in the case of COVID-19 pandemics, GT was used to assess public interest in various medical and biomedical fields [14], [15], [16], [17], [18]. It should be noted that evaluating public interest in vaccination against COVID-19 during pandemics indicates knowledge gaps. So, information retrieved from GT may be used to determine whether people have access to the information about COVID-19 vaccines and guide policymakers and healthcare providers on addressing the public information demands during the COVID-19 pandemic. Therefore, this study aimed to use the GT data to assess the global public interest in coronavirus vaccines during the pandemic.

## Materials and methods

To assess the status of inquiries related to the coronavirus vaccine, we used “Worldwide” as the search range, “01/19/2020-07/04/2021” as the time range, ”All categories“ as the category, and ”Web Search“ as the search type. The English terms were selected after searching Naver (the Korean search engine) where we evaluated their popularity. The final selection included the following 39 keywords or possible combinations: *Corona Vaccine, Corona Vaccine Development, COVID-19 Vaccine Development, China Corona Vaccine, Corona Chinese Vaccine, US Corona Vaccine, UK Corona Vaccine, Korea Corona Vaccine, Russian Vaccine, Sputnik v, Moderna Vaccine, Pfizer Corona Vaccine, AstraZeneca Vaccine, 2019 Novel Coronavirus Disease, 2019 Novel Coronavirus Infection, 2019 Novel Coronavirus, 2019nCoV Disease, 2019nCoV Infection, 2019nCoV, 2019nCoV-co, Coronavirus Disease 2019 Virus, Coronavirus Disease 2019, Coronavirus Disease-19, Covid 19, COVID19 Virus, COVID-19 Virus, COVID19, COVID-19, COVID-2019, SARS2, SARS-CoV-2, Vaccina, Vaccine, Vaccines, Vaccination, Wuhan Corona Virus, Wuhan Seafood Market Pneumonia Virus, Severe Acute Respiratory Syndrome Coronavirus 2, Coronavirus.*

We tracked overall interest in keywords on the Internet and then compared interest in terms by country over a selected period. The data is downloaded from the Internet in “.csv” format. The countries with the highest volume of queries always have the highest rankings. The number of queries received is scaled from 0 to 100, depending on the topic's relationship to all searches across all topics. GT uses a relative popularity metric, meaning that the maximum number of queries for the selected period will always be 100. Google's normalized trend data is a useful metric for assessing the popularity of topics as measured by the number of published medical studies. The number of publications was assessed using the PubMed service with the same keywords selected earlier.

## Results

**Fig. 1:**
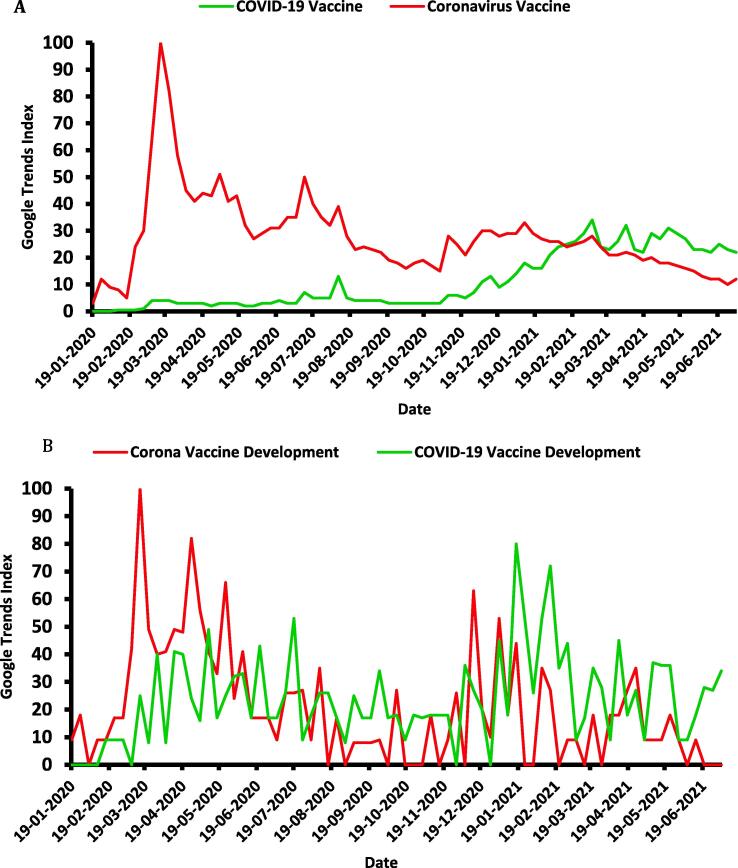
A, the total relative number of requests in the world for *COVID-19 Vaccine*, *Coronavirus Vaccine* for the period from Jan 19, 2020, to Jul 04, 2021; B, the total relative number of requests in the world in terms of *Corona Vaccine Development*, *COVID-19 Vaccine Development* for the period from Jan 19, 2020, to Jul 04, 2021.

From August 1st till December 31st, 2020, the relative search volume for 21 of the 31 search terms significantly increased compared to the period from February 1st till July 31st, 2020. However, nine search terms showed a significant increase in the same period (Table 1). From Jan 19, 2020, to Jan 19, 2021, the relative search of 31 keywords showed a significant increase in the trends of the number of global requests. The dynamics of weekly recommendations showed a substantial increase in interest in *COVID-19 Vaccine* and *COVID-19 Vaccine Development* (Fig. 1**A, 1B**). However, for terms like the *Coronavirus Vaccine*, the *Development of a Coronavirus Vaccine*, the dynamics are opposite (Fig. 1**A, 1B**).

**Table 1:** Relative search volume of COVID-19 and vaccine.

**Search terms**	**Time courses**			
	**Feb 1**, **2020 – July 31, 2020**	**August 1**, **2020 – Dec 31, 2020**	**% Change**	***P* value**
2019 novel coronavirus disease	20.81 ± 10.983	16.02 ± 5.169	−4.79	<0.001
2019 novel coronavirus infection	11.62 ± 6.371	22.09 ± 5.721	10.47	<0.001
2019 novel coronavirus	22.42 ± 10.867	29.36 ± 0.69	6.94	<0.001
2019nCov	10.45 ± 6.632	19.275 ± 4.28	8.825	<0.001
Coronavirus disease 2019 virus	14.81 ± 5.292	19.54 ± 4.37	4.73	<0.001
Coronavirus disease 2019	11.09 ± 7.576	58.82 ± 7.32	47.73	<0.0001
covid 19	20.96 ± 4.879	60.47 ± 7.67	39.51	<0.0001
COVID19 virus vaccine	27.68 ± 4.526	56.15 ± 7.11	28.47	<0.0001
COVID-19 virus vaccine	30.47 ± 6.650	36.50 ± 1.20	6.03	<0.001
COVID19 virus	20.96 ± 4.879	41.452 ± 7.51	20.49	<0.0001
COVID-19 virus	27.69 ± 4.26	44.90 ± 2.96	17.21	<0.0001
COVID19	24.33 ± 9.160	63.04 ± 6.83	38.71	<0.0001
COVID-2019	14.64 ± 4.819	30.30 ± 4.579	15.66	<0.0001
SARScov2	48.14 ± 4.472	73.51 ± 5.88	25.37	<0.0001
Vaccina	30.96 ± 6.835	47.01 ± 9.73	16.05	<0.001
Vaccine	48.42 ± 8.487	30.77 ± 2.03	−17.65	<0.0001
vaccination	62.71 ± 2.057	20.68 ± 4.05	−42.03	<0.0001
Vaccines	59.37 ± 6.346	51.19 ± 1.95	−8.18	<0.001
Wuhan corona virus	28.32 ± 6.746	42.95 ± 1.13	14.63	<0.0001
Wuhan seafood market pneumonia virus	21.071 ± 5.31	10.1 ± 0.96	−10.97	<0.0001
Severe acute respiratory syndrome coronavirus 2	29.69 ± 4.482	25.51 ± 2.43	−4.18	<0.001
AstraZeneca vaccine	12.097 ± 6.98	14.89 ± 4.76	2.793	0.0003
China corona vaccine	19.91 ± 6.526	24.11 ± 1.20	4.20	<0.001
Corona Chinese vaccine	21.66 ± 5.99	22.58 ± 2.51	0.92	0.1350
Korea Corona Vaccine	17.454 ± 0.85	21.13 ± 0.35	3.676	<0.001
Moderna vaccine	10.974 ± 3.28	17.93 ± 3.97	6.956	<0.001
Pfizer Corona Vaccine	18.719 ± 3.32	11.039 ± 0.97	−7.680	<0.001
Russian corona vaccine	9.926 ± 2.53	13.14 ± 0.76	3.214	<0.001
Sputnik v	24.233 ± 4.48	10.24 ± 0.56	−13.993	<0.001
UK corona vaccine	16.49 ± 5.81	11.43 ± 0.80	−5.06	<0.001
US Corona Vaccine	10.223 ± 7.85	19.25 ± 0.48	9.27	<0.001

**Fig. 2:**
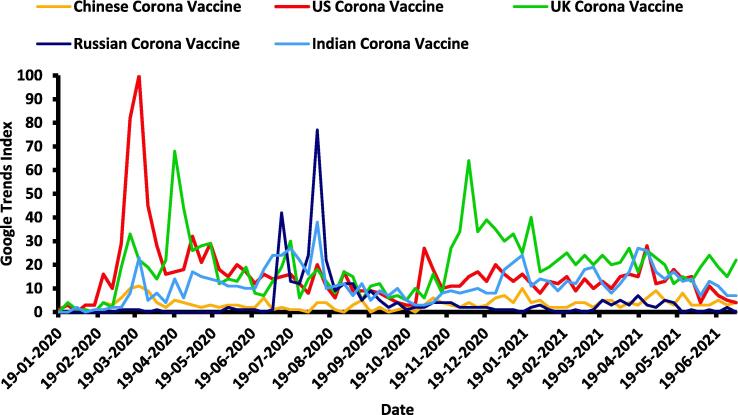
The total relative number of requests in the world for the terms: *Chinese Corona Vaccine*, *US Corona Vaccine, UK Corona Vaccine, Russian Corona Vaccine, Indian Corona Vaccine*, from Jan 19, 2020, to Jul 04, 2021.

Considering the type of Covid-19 vaccines, the period from Jan 2020 to Jun 2021 showed a stable interest in all vaccines (especially *US Corona Vaccine, UK Corona Vaccine, Indian Corona Vaccine*) periodically observed surges of interest. For example, an increased interest in *Russian Corona Vaccine* was manifested in the summer of 2020, and interest in *UK Corona Vaccine* grew in the winter of 2020–2021 (Fig. 2). Demands for the *Korean Corona Vaccine* were not significant (not shown in the figure).

We have analyzed requests for vaccines where users indicate the country of origin/vaccine development.

We compared the number of requests for a *Chinese Corona Vaccine* in different formulations. The analysis shows that the more popular query includes the name of the country followed by the terms *Corona Vaccine*. Further, we formulated requests in this way (Fig. S1).

Total (100%) of the *China Corona Vaccine* requests come from countries such as Zimbabwe, Kuwait, Singapore, Malaysia, Portugal, Egypt, Brasil, Mexico, and France.

**Fig. 3:**
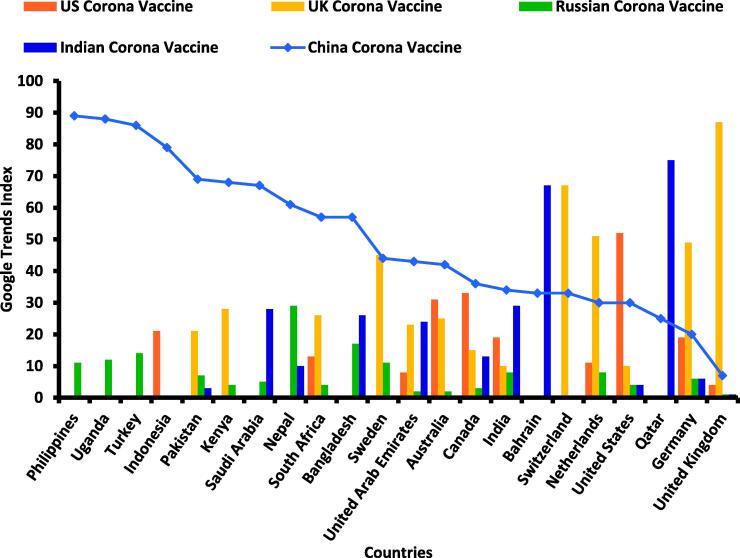
The relative number of requests for *China Corona Vaccine, US Corona Vaccine, UK Corona Vaccine, Russian Corona Vaccine, Indian Corona Vaccine* by countries interested in more than two vaccines (from Jan 19, 2020, to Jul 04, 2021).

Total (100%) of the *Russian Corona Vaccine* requests come from Russia. After excluding countries that are interested in a vaccine of only one origin, we got Fig. 3**.**

**Fig. 4:**
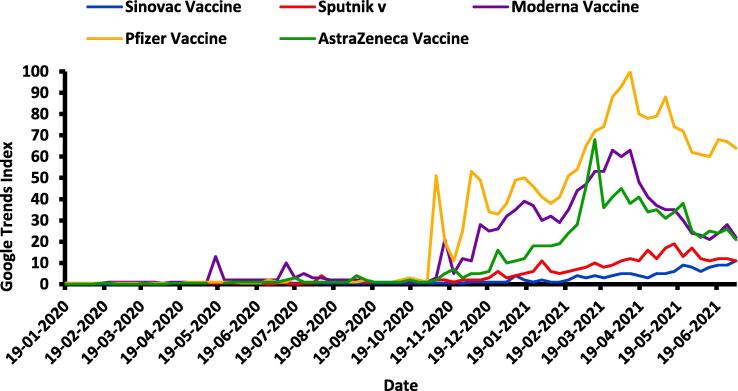
The total relative number of worldwide requests from Jan 19, 2020, to Jul 04, 2021.

The annual trend analysis revealed an upward trend for most of the branded vaccines reviewed (Fig. 4).

To calculate the trends, a shorter time was used: from May 24, 2020, to Jul 04, 2021 (from the date when at least one value became nonzero). A polynomial growth is noted for *Sputnik v* (y = 0,0001x^2^ − 9,2086x + 202549, R^2^ = 0,8462), *Sinovac Vaccine* (y = 0,0001x^2^ − 9,1206x + 201039, R^2^ = 0, 9148). For *AstraZeneca Vaccine* there was a power-law trend growth (y = 7E−283e^0,0148x^, R^2^ = 0,815), for *Pfizer Vaccine* there was exponential growth (y = 7E−283e^0,0148x^, R^2^ = 0,815). For *Moderna Vaccine* there was exponential growth too (y = 3E−188e^0,0098x^, R^2^ = 0,6468).

**Fig. 5:**
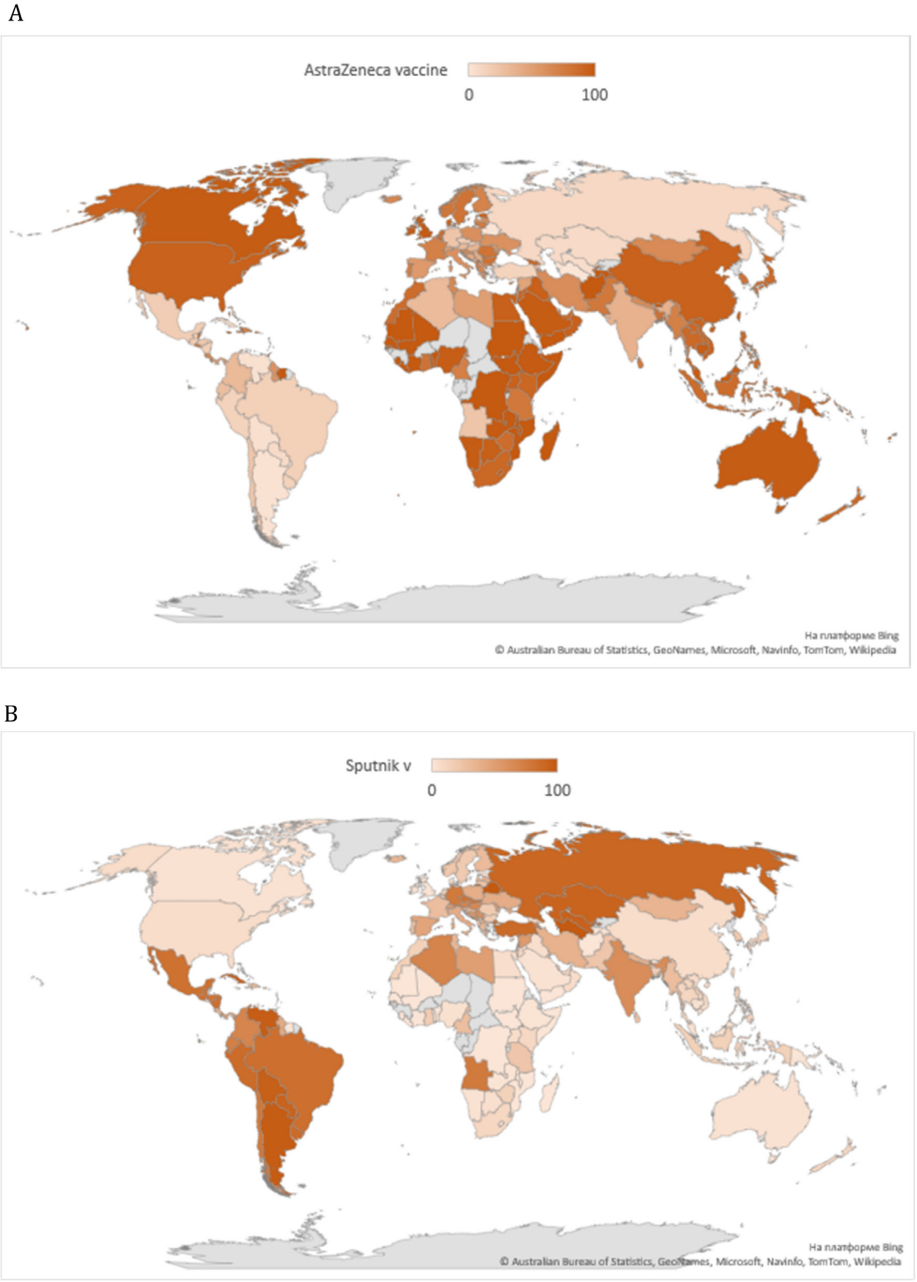
Search queries as a percentage of the total number of queries in the countries from Jan 19, 2020, to Jul 04, 2021 (when considering two types of queries *Sputnik v, AstraZeneca Vaccine*). **A**, the global interest in *AstraZeneca Vaccine*. **B**, the global interest in *Sputnik v* vaccine.

We looked at the interest in vaccines *Sputnik v* and *AstraZeneca* by countries (Fig. 5).

In countries such as Turkmenistan and Belarus, the users were interested only in the *Sputnik v* vaccine. A high level of interest in the *Sputnik v* vaccine (more than 90% of requests) was observed in countries such as Argentina, Venezuela, Bolivia, Cuba, Uzbekistan, Kazakhstan, Paraguay, Russia (listed in descending order of the number of requests from 98 to 91%).

Countries like Fiji, Barbados, Malta, Jamaica, St Kitts & Nevis, Solomon Islands, Anguilla, Antigua & Barbuda, Vanuatu, British Virgin Islands, Isle of Man, St Lucia, Papua New Guinea, San Marino, Timor-Leste, Grenada, Malawi, Bahamas, Belize, Guernsey, Dominica, Suriname, Bermuda, Jersey, Brunei, Somalia, Bhutan, Rwanda, Seychelles, Eswatini, Sint Maarten, Faroe Islands, Gambia, South Sudan, Lesotho, Gibraltar, Sierra Leone, Andorra, Aruba, Liberia, Cayman Islands, Sudan, Togo, Afghanistan, Madagascar, Senegal, Mali, Guam, Haiti, Macao, Mozambique, Mauritania, Congo – Kinshasa, Tajikistan, Cote d'Ivoire, Benin, Yemen were only interested in the *AstraZeneca Vaccine*.

More than 90% of search queries were for this vaccine in countries such as Trinidad & Tobago, United Kingdom, Ethiopia, Zambia, Ireland, Canada, Namibia, Australia, Saudi Arabia, Malaysia, Cambodia, Nigeria, Botswana, Kuwait, St Helena, St Vincent & the Grenadines, Denmark, Egypt, Iraq, Oman, Uganda, United States, China, Kosovo, Vietnam, Maldives, Singapore, Estonia, Mauritius, Kenya, Jordan, Cyprus, South Africa, New Zealand, Taiwan (listed in descending order of the number of requests from 99 to 90%).

Fig. 5 shows that these vaccines are competitors.

We reviewed the publication activity for the keywords *COVID-19 vaccine, Coronavirus vaccine* according to PubMed monthly data for 2020 (Fig. S2). The correlation was calculated between the monthly number of keywords’ searches and the monthly number of publications. For *COVID-19 vaccine* R = 0.3357, for *Coronavirus vaccine* R = 0.0758. Consequently, the correlation between scientific research and user queries is absent.

To get an idea of the population's attitude to the coronavirus vaccine, we reviewed the results of search queries for the terms *COVID-19 Vaccine Side Effects*, *COVID-19 Vaccine Good*, *COVID-19 Vaccine Dangerous*, *COVID-19 Vaccine Safe* (Fig. S3). It turned out that users are mainly interested in the side effects of the vaccine. Fig. S4 shows that users in many countries have focused on finding out the side effects of the coronavirus.

## Discussion

The present study aims to use the GT data to assess the global public interest in COVID-19 vaccines during the pandemic. This study showed a statistically significant increase in the relative search volume of more than half of the search terms from Jan 19, 2020, to Jul 04, 2021. Previous studies assessed the public interest of various medical disciplines during COVID-19 pandemics using GT and reported an increase in relative search for the terms of interest [19], [20], [21], [22], [23]. Other studies evaluated the global public interest in different surgical and interventional procedures during COVID-19 pandemics using GT. They showed a decrease in relative search for the related terms, representing a fear of surgical intervention among the general population during the COVID-19 pandemic [17], [24], [25], [26], [27], [28], [29], [30], [31], [32]. Other groups of researchers studied the public's interest in the use of telehealth, dietary supplements, the geographical distribution of the severity of the disease, and possible treatments during the COVID-19 pandemic [33], [34], [35], [36], [37], [38], [39], [40], [41]. Consequently, all studies have shown a significant increase in relative search volume for most of the search terms in the COVID-19 pandemic period [40]. However, they have also demonstrated a shift of public interest from the COVID-19 disease to vaccines.

From Jan 19, 2020, to Jan 19, 2021, relative searches for 31 keywords showed a significant upward trend in the number of global searches for terms such as *Coronavirus Disease 2019* (+47.73%), *Covid 19* (+39.51%), *COVID19* (+38.71%), *COVID19 Virus* (+20.49%). In addition, the number of requests for the term *COVID19 Virus Vaccine* increased significantly (+28.47%). The dynamics of weekly recommendations showed a significant increase in interest in the terms *COVID-19 Vaccine* and *COVID-19 Vaccine Development.* On the contrary, the number of requests decreased significantly for the *Coronavirus Vaccine* for the *Development* of a *Coronavirus Vaccine*, the number of requests decreased significantly. This is due to the growing awareness of the population about the disease and related terminology.

There has been a significant increase in the number of requests globally for the terms *Coronavirus Vaccine* from China, USA, UK, Russia, Korea from February 2020 to March 2020, when WHO announced the coronavirus pandemic on March 11. A surge of interest in the Russian vaccine was noted in July-August. In early August, the registration of the world's first coronavirus vaccine was announced in Russia. The growing interest in the vaccine from the UK is since the authorities announced the start of vaccination against coronavirus in early December.

We analyzed vaccine requests where users indicate the country of origin of vaccine development. The example of China shows that the more popular query is the one that includes the name of the country followed by the terms. The geographical distribution of requests for national vaccines was reviewed. It turned out that only the Chinese vaccine is interested in such countries as Kuwait, Singapore, Malaysia, Portugal, Egypt, Brazil, Mexico, France, and Zimbabwe. Only South Korea is interested in the Korean vaccine. 87% of requests for a UK vaccine came from the United Kingdom. Overall, 52% of recommendations for a vaccine from the United States came from the United States, that is, Americans are more interested in vaccines from other countries. Belarus and Turkmenistan are exclusively interested in the *Sputnik v* vaccine. This vaccine prevails in the requests from the countries: Argentina (98%), Venezuela (97%), Bolivia (96%), Cuba (95%), Uzbekistan (94%), Kazakhstan and Paraguay (93%), and Russia (91%).

Users of countries of Barbados, Malta, Jamaica, St Kitts & Nevis, Anguilla, Antigua & Barbuda, British Virgin Islands, Isle of Man, St Lucia, San Marino, Timor-Leste, Grenada, Bahamas, Belize, Guernsey, Dominica, Suriname, Bermuda, Jersey, Brunei, Somalia, Bhutan, Rwanda, Seychelles, Eswatini, Sint Maarten, Faroe Islands, Gambia, South Sudan, Gibraltar, Sierra Leone, Andorra, Aruba, Liberia, Cayman Islands, Sudan, Togo, Afghanistan, Madagascar, Senegal, Mali, Guam, Haiti, Macao, Mozambique, Mauritania, Tajikistan, Yemen, some countries of Oceania and Africa are exclusively interested in the *AstraZeneca Vaccine*. This vaccine dominates among user requests from Trinidad & Tobago, United Kingdom, Ethiopia, Zambia (99%), Ireland, Canada, Namibia, Australia, Saudi Arabia (98%), Malaysia, Cambodia, Nigeria (97%), Botswana, Kuwait, St Helena (96%), St Vincent & the Grenadines, Denmark, Egypt, Iraq (95%), Oman, Uganda, United States, China (94%), Kosovo, Vietnam (93%), Maldives, Singapore, Estonia (92%), Mauritius, Kenia, Jordan (91%), and Cyprus, South Africa, New Zealand, Taiwan (90%).

For Sputnik v and Sinovac vaccines, a polynomial increase in the number of requests was noted. For Pfizer and Moderna vaccines, an exponential increase in the number of requests was noted. For the AstraZeneca vaccine, there was a power-law trend growth.

Comparison of publication activity with the number of requests showed no correlation between scientific research and user requests. The analysis showed that users from United Arab Emirates, Singapore, Ireland, South Africa, Israel, Pakistan, Saudi Arabia, and Malaysia were exclusively interested in the vaccine's side effects. User interest in vaccine side effects prevailed in countries such as the United Kingdom (74%), the United States (69%), Australia (64%), Philippines (57%), and Canada (56%). Users from India were the most supportive of the vaccine, with 55% searching for the term *COVID-19 Vaccine Good*.

WHO has launched an international campaign entitled “Managing the COVID-19 infodemic: Promoting healthy behaviors and mitigating the harm from misinformation and disinformation” to draw the attention of governments to counter the spread of misinformation. One of the main current concerns is the spreading of unconfirmed data about vaccines and vaccination, which could seriously undermine the international strategy to combat SARS-CoV-2. Therefore, the need for new effective and efficient infodemiological methods is more urgent than ever. The relationship between Internet inquiries, media reports, and evidence of morbidity is multifactorial and requires further study. Nevertheless, the main trends in Internet search queries during a pandemic can serve as an additional component of epidemiological surveillance.

Rovetta [41] demonstrated that GT has limitations that are often overlooked and can severely bias and distort correlation-based analytics. The author showed the dependence of the relative search volumes (RSV) GT values on the date of their collection. The actual algorithm by which GT discovers the query data is currently unknown. Nevertheless, GT serves as a valuable tool for information surveillance and infodemiology. The effectiveness of GT can be crucial in the epidemiological and infodemiological assessment of the web interests of users around the world. GT can give statistical estimates of the topic information flow. This article is the starting point for further analysis of the information related to coronavirus vaccination.

## Limitations

Our research used GT, which shows the search behavior of people using the Google search engine. Future research should consider exploring the same topic on a different search engine including social networks to reach a more diverse user audience.

While analyzing the number of requests, we deal not with the absolute, but with the relative number of requests. In addition, this tool does not consider repeated searches from the same user in a short period, distorting the results' objectivity. The limitation of the study is the lack of GT data about China because of the general unavailability of Google in China.

## Conclusions

Interest in vaccines produced by different countries and companies varied in time and space. For example, the bursts of interest were timed to the announcement of the vaccine registration in Russia (August 2020), and to the announcement of the start of vaccination in the UK (December 2020). There was a geographical differentiation of interest in vaccines produced by a specific country or pharmaceutical company. All applied vaccines are of particular interest to users. We were investigating the use of search engines to gauge user interest in COVID 19 vaccines. Our results demonstrated the potential for using GT as a complementary tool to help understand Internet searches that can determine the demand for vaccines from different countries or companies and the geographic distribution of these demands.

This study shows that Internet search patterns can be used to gauge public attitudes towards coronavirus vaccination. Interest in vaccine side effects is consistently high, followed by safety concerns. The flow of inquiries about side effects especially increased in December, when the vaccination company began in Russia, Germany, France, Spain, Austria, Bulgaria, Czech Republic, Italy, Turkey, and other countries. This data can be used to track and predict attitudes towards vaccination of populations from COVID-19 in different countries before widespread vaccination becomes available to help mitigate the adverse effects of the pandemic.

The search queries can reflect the interests of users in the field of unreliable information. For example, the testing period for coronavirus vaccines is not yet very long; therefore, due to a lack of knowledge, fakes arise (for instance, infertility after vaccination). In addition, the information provided to the public can be politically charged. Statistical processing of information shows more reliable results with an increase in the amount of processed data.

## Declaration of Competing Interest

The authors declare that they have no known competing financial interests or personal relationships that could have appeared to influence the work reported in this paper.
