# Supplementary material for: Global interest in vaccines during the COVID‐19 pandemic: Evidence from Google Trends
Source: Vaccine X. 2022 Mar 11;10:100152. doi: 10.1016/j.jvacx.2022.100152 (PMC8915451; doi:10.1016/j.jvacx.2022.100152)

**Supplementary 2**

**Figure S1:** Co-authorship network for «vaccine + coronavirus» publications 2019-2020 on the A, Scopus database; B, PubMed database; Dimensions database (by organizations)


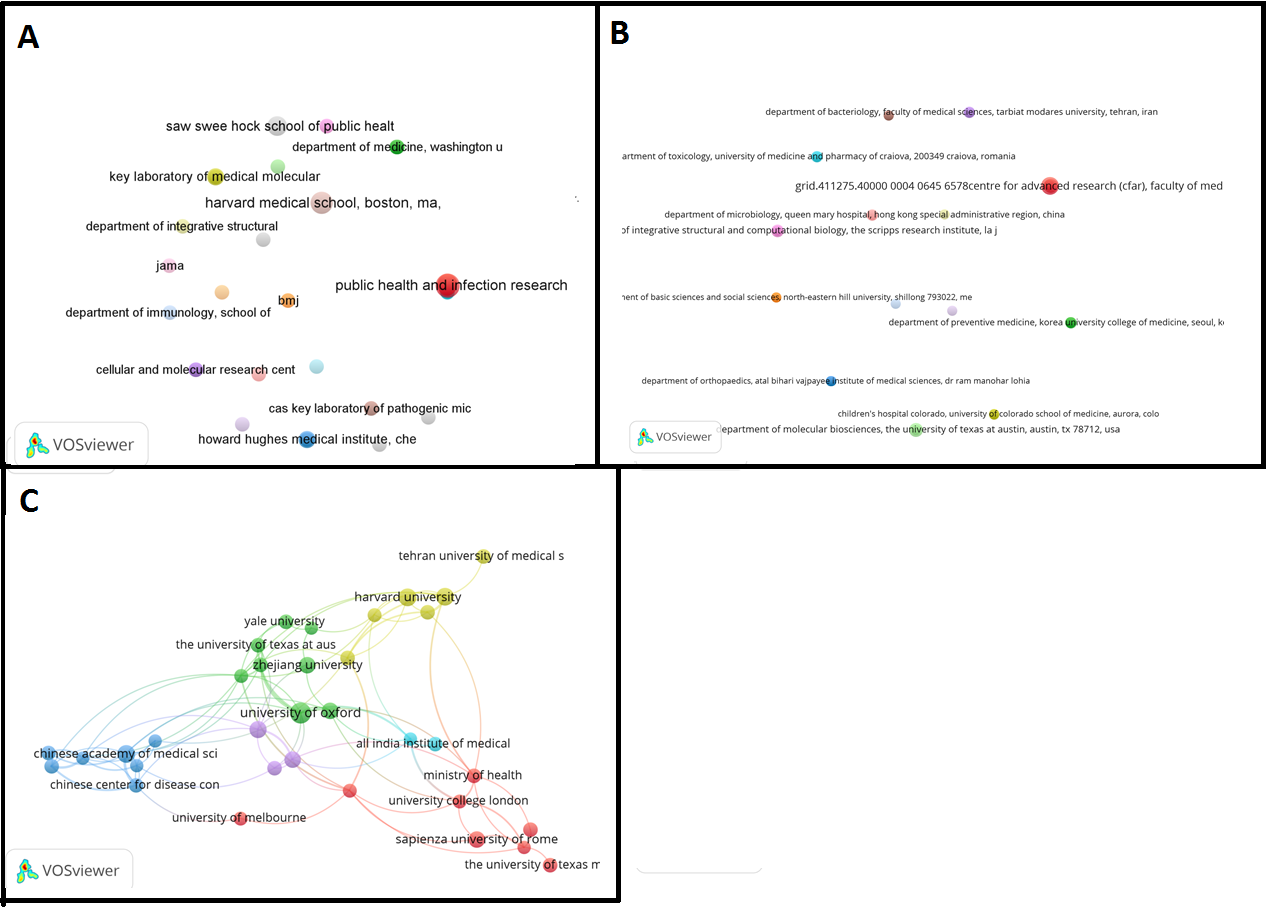


**Figure S2**. Co-occurrence network map of 39 keywords for «vaccine + coronavirus» publications 2019-2020 on the A, Scopus database; B, PubMed database; C, Dimensions database


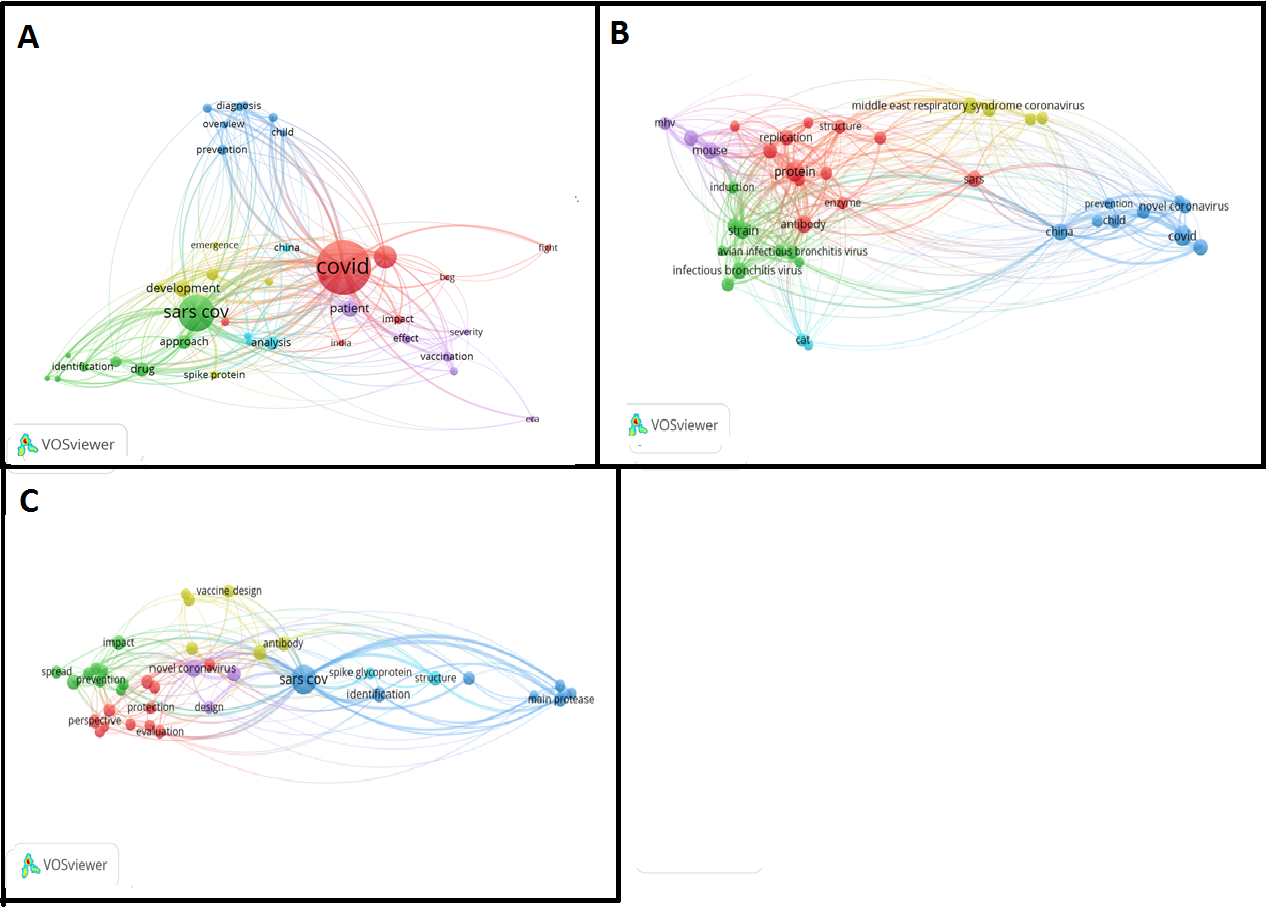


**Figure S3**. The comparison of key terms from different corpora (Scopus, PubMed, Dimensions)


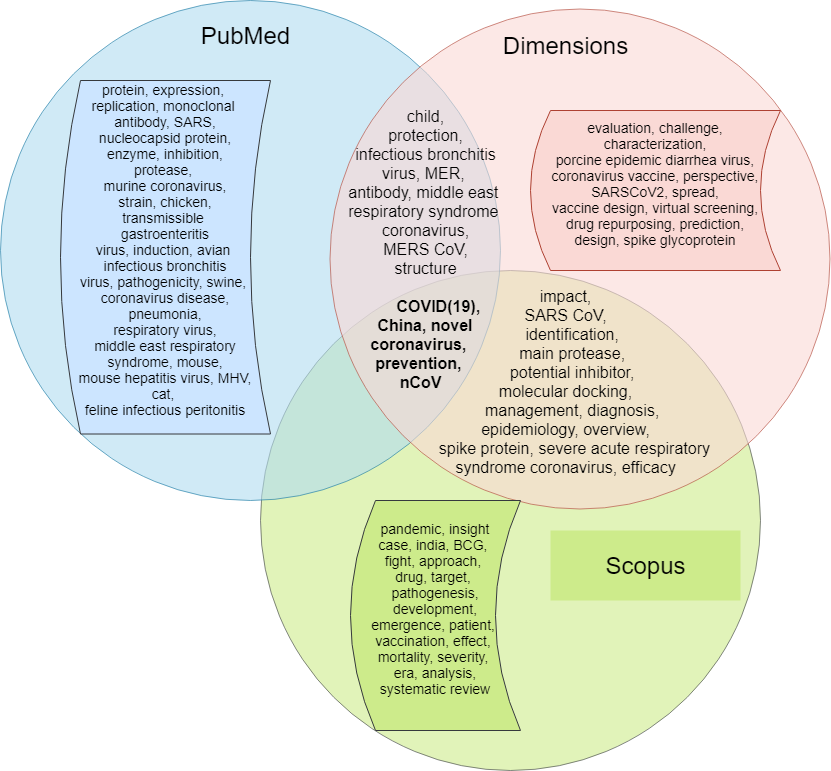


**Figure S4.** Co-occurrence network map of 475 keywords for «vaccine + coronavirus» publications 2019-2020 on the A, Scopus database; B, PubMed database; C, Dimensions database


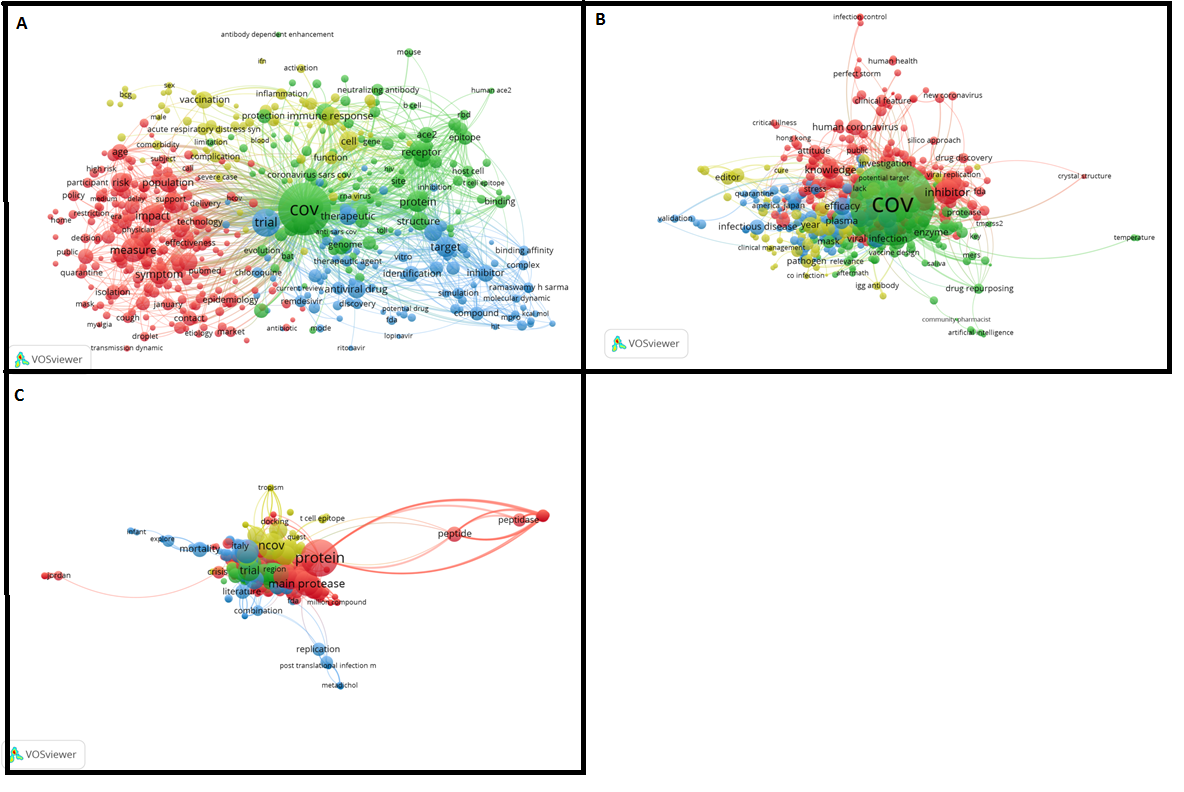

Supplement: Supplementary data 1 [file mmc1.docx]
